# Usability and Preliminary Efficacy of an Adaptive Supportive Care System for Patients With Cancer: Pilot Randomized Controlled Trial

**DOI:** 10.2196/49703

**Published:** 2024-07-10

**Authors:** Sharon H Baik, Karen Clark, Marisol Sanchez, Matthew Loscalzo, Ashley Celis, Marianne Razavi, Dershung Yang, William Dale, Niina Haas

**Affiliations:** 1 Department of Supportive Care Medicine City of Hope Duarte, CA United States; 2 BrightOutcome Buffalo Grove, IL United States

**Keywords:** cancer, distress screening, eHealth, supportive care, mobile phone

## Abstract

**Background:**

Using an iterative user-centered design process, our team developed a patient-centered adaptive supportive care system, PatientCareAnywhere, that provides comprehensive biopsychosocial screening and supportive cancer care to patients across the continuum of care adaptively. The overarching goal of PatientCareAnywhere is to improve health-related quality of life (HRQOL) and self-efficacy of patients with cancer by empowering them with self-management skills and bringing cancer care support directly to them at home. Such support is adaptive to the patient’s needs and health status and coordinated across multiple sources in the forms of referrals, education, engagement of community resources, and secure social communication.

**Objective:**

This study aims to assess the usability of the new web-based PatientCareAnywhere system and examine the preliminary efficacy of PatientCareAnywhere to improve patient-reported outcomes compared with usual care.

**Methods:**

For phase 1, usability testing participants included patients with cancer (n=4) and caregivers (n=7) who evaluated the software prototype and provided qualitative (eg, interviews) and quantitative (eg, System Usability Scale) feedback. For phase 2, participants in the 3-month pilot randomized controlled trial were randomized to receive the PatientCareAnywhere intervention (n=36) or usual care control condition (n=36). HRQOL and cancer-relevant self-efficacy were assessed at baseline (preintervention assessment) and 12 weeks from baseline (postintervention assessment); mean differences between pre- and postintervention scores were compared between the 2 groups.

**Results:**

Participants were highly satisfied with the prototype and reported above-average acceptable usability, with a mean System Usability Scale score of 84.09 (SD 10.02). Qualitative data supported the overall usability and perceived usefulness of the intervention, with a few design features (eg, “help request” function) added based on participant feedback. With regard to the randomized controlled trial, patients in the intervention group reported significant improvements in HRQOL from pre- to postintervention scores (mean difference 6.08, SD 15.26) compared with the control group (mean difference −2.95, SD 10.63; *P*=.01). In contrast, there was no significant between-group difference in self-efficacy (*P*=.09).

**Conclusions:**

Overall, PatientCareAnywhere represents a user-friendly, functional, and acceptable supportive care intervention with preliminary efficacy to improve HRQOL among patients diagnosed with cancer. Future studies are needed to further establish the efficacy of PatientCareAnywhere as well as explore strategies to enhance user engagement and investigate the optimal intensity, frequency, and use of the intervention to improve patient outcomes.

**Trial Registration:**

ClinicalTrials.gov NCT02408406; https://clinicaltrials.gov/study/NCT02408406

## Introduction

### Background

One-third [1,2] to half [3-5] of patients with cancer report psychological distress. Common causes of distress include fatigue, pain, worry about the future, finances, and the side effects of treatment [6-8]. Supportive care is a complex specialty that encompasses an array of multidisciplinary services addressing a variety of biopsychosocial concerns and needs. The 2008 Institute of Medicine report, *Cancer Care for the Whole Patient* [6], lists the main supportive care services as “information about illness, treatments, health, and services; help in coping with emotions accompanying illness and treatment; help in managing illness; assistance in changing behaviors to minimize impact of disease; material and logistical resources, such as transportation; help in managing disruptions in work, school, and family life; and financial advice and/or assistance.” In addition to these formal sources of supportive care, the report stressed that informal sources, such as family and friends, are also key providers of supportive care. At the heart of a successful supportive care practice is comprehensive biopsychosocial screening, covering multiple domains including physical symptoms, psychosocial issues, and practical concerns. Effective biopsychosocial screening integrated with triage, referrals, patient and caregiver education, and follow-up services promotes successful whole patient-centered care across the cancer treatment trajectory. Studies have demonstrated that adequate integration of biopsychosocial screening with supportive care results in better patient outcomes [9-18], better patient-provider communication [9,12,15,19-25], higher patient satisfaction [12,20,22-24], detection of unrecognized problems [10,12,15,21,23-25], improved referrals [11,25-29], and better health service use and lower costs [30-35].

Recognizing the importance of distress management, the National Comprehensive Cancer Network (NCCN) recommends distress screening for all patients with cancer to address problems before a crisis develops and necessitates higher levels of intervention, with guidelines in place since 1999 [36]. Unfortunately, a serious gap remains between the screening services that are needed and those provided today [6,37,38]. In a 2018 survey to NCCN member institutions, 87% (20/23) of institutions reported conducting routine screening for distress as per the guidelines, but only 26% (6/23) strived to screen all patients and 57% (13/23) screened outpatients only [39]. Compared with the 2012 survey [37], the percentage of institutions conducting screening of all patients decreased from 30% to 26% and the percentage of institutions screening outpatients only increased from 50% to 57% over a 6-year span. Most institutions administered screening via paper and pencil (12/23, 52%) or electronically (12/23, 52%), while 30% conducted interviews (6 in person and 1 via telephone). In addition, only 7 institutions reported automatic triage based on computer-generated results, whereas 14 institutions required clinical staff to manually review the screening results to generate referrals.

Furthermore, there is often a large gap between the onset of patients’ distress and the communication about it to their health care team, especially when these problems and symptoms occur outside of the clinical environment. This disconnect is exacerbated by the lack of uniform systems to document problems and communications between the health care professionals themselves. In addition, the absence of systematic criteria-based identifiers for referring patients to suitable consultation services and resources results in important clinical information not being communicated promptly to the appropriate professionals. Electronic methods for distress screening, including automated touch screen technologies and web-based assessments, have been recommended as they can be helpful with systematically identifying, tracking, and managing sources of distress [40]. Over the past decade, technology and eHealth interventions have increasingly been used in the delivery of patient-centered cancer care [41-43]. A recent systematic review of technology-based supportive care interventions for patients with cancer demonstrated significant effects on health-related quality of life (HRQOL), cancer-related symptoms, levels of fatigue and pain, depression, and functional capacity [44]. A meta-analysis was precluded due to heterogeneity in intervention design and features (eg, duration, frequency, and use of technology) and outcome measures.

To address this pressing gap in supportive cancer care, City of Hope in partnership with BrightOutcome, a health care technology company, developed a technology-based, patient-centered adaptive supportive care system for patients newly diagnosed with cancer (named PatientCareAnywhere) using an iterative user-centered design process. PatientCareAnywhere was derived from two existing systems: (1) SupportScreen from City of Hope [45], a clinic-based biopsychosocial screening tool that connects new patients with individualized educational and professional symptom triage support based on self-reported distress; and (2) MyCaringCircle from BrightOutcome, a home- and community-based patient portal solution that offers self-reported symptom assessment, individualized education content delivery, facilitation of remote medical care, and coordination of support from the patient’s friends and family and from community resources. While SupportScreen excels in the provision of a broad range of biopsychosocial screenings, facilitation of referrals, and integration of electronic health records (EHRs), MyCaringCircle’s strengths are its focus on symptom assessment via its access to a large library of validated measures and its facilitation of social support outside the clinical environment involving community resources.

### Objective

This study includes 2 phases. In phase 1, with the software prototype, we conducted usability tests, which are an integral part of the user-centered design process and help ensure the intervention meets users’ expectations and functions as intended. In phase 2, to evaluate the preliminary efficacy of PatientCareAnywhere compared with usual care (control condition), we conducted a pilot randomized controlled trial (RCT) evaluating changes in self-reported patient outcomes, including HRQOL and self-efficacy, from baseline to postintervention assessment. We hypothesized that PatientCareAnywhere would result in significant improvements in HRQOL and patient self-efficacy compared with usual care among patients newly diagnosed with cancer.

## Methods

### PatientCareAnywhere

#### Overview

City of Hope, in partnership with BrightOutcome, a health care technology company, developed a patient-centered adaptive supportive care system (PatientCareAnywhere) to improve patient outcomes for patients with cancer while reducing health care costs. This project was funded by the National Cancer Institute via a Small Business Innovation Research Fast-Track grant (R44CA192588). PatientCareAnywhere is a patient empowerment solution that promotes internal resilience, self-efficacy, and independence. The key features of PatientCareAnywhere include (1) multilevel and adaptive biopsychosocial screening covering a comprehensive set of supportive cancer care domains (eg, emotional, physical, practical, and social) without overburdening patients with long static questionnaires; (2) automatic alert messages for abnormal screening results to clinical team; (3) specialist referrals and community support resources based on screening results; (4) individualized patient education contents based on screening results; (5) social media support for engagement of caregivers, family, friends, and community resources; (6) optimized display for different devices (eg, smartphones and tablets); and (7) EHR integration. The PatientCareAnywhere experience begins with an initial comprehensive biopsychosocial assessment covering physical symptoms (eg, pain), psychosocial issues (eg, anxiety), and practical concerns (eg, finances). Table 1 provides a list of biopsychosocial screening topics and designated care professionals for follow-up. The assessments start with first-level questions, which, when a patient’s response exceeds a pre-established threshold, will trigger additional follow-up questions to gain further insights into the patient’s needs and concerns. Additionally, alert messages are generated for the clinical and support care teams.

These self-reported needs and the individual’s disease and treatment stages form the basis for PatientCareAnywhere to offer responsive supportive care in terms of individualized patient education content, triage to specialists, and referrals to community resources. Cancer-specific content (eg, information about breast, lung, or prostate cancer and its treatment) and generic content (eg, emotional distress) was adapted from public domain sources, such as the National Cancer Institute, American Cancer Society, and NCCN, and from materials developed by the Division of Patient and Family Community Education at City of Hope. We also collected contact information for supportive care services provided by City of Hope and local community resources, which were recommended to patients based on their self-reported symptoms and needs. The system was designed to be used by patients, friends and families, health care professionals, and community resources. With PatientCareAnywhere, patients are at the center of the “circle of care,” receiving support from multiple clinical, social, and community sources and across the continuum of care, from diagnosis to treatment to survivorship and end-of-life care. In addition, PatientCareAnywhere provides a communication platform to allow caregivers, family members, and friends to interact directly with the patient through the system. As a security feature, patients have complete control over who is included in their care circles and how much communication or information is shared with each person invited. In particular, caregivers are granted full access to patient medical records and can obtain information about the patient’s current medications, laboratories and tests, vitals, biopsychosocial screenings, and symptom histories as well as keep track of medical appointments on PatientCareAnywhere, while noncaregivers have limited access. The main components of PatientCareAnywhere are listed in Multimedia Appendix 1, and screenshots of PatientCareAnywhere are included in Multimedia Appendix 2.

**Table 1:** Biopsychosocial screening items and associated referrals.

Biopsychosocial screening items		Primary follow-up
**Social and practical needs**		
	Ability to have children	Physician
	Communication about medical care	Pharmacy clinical manager, physician, social worker
	Finding local support resources	Social worker
	Finding reliable medical information	Cancer information resource nurse, nurse
	Health insurance	Financial counselor
	Help with home or medical care	Patient navigator, resource coordinator, social worker
	Hospice service	Physician, nurse practitioner or physician extender
	Personal finances	Social worker
	Physical appearance	Positive image center, social worker
	Social support	Social worker
	Spiritual or religious concerns	Chaplin
	Worries about the future	Social worker
**Physical and emotional well-being**		
	Anxiety	Social worker
	Appetite loss	Nurse
	Bladder control	Nurse
	Breathing difficulties	Nurse
	Bowel control	Nurse
	Cognitive issues	Physician
	Constipation	Nurse
	Depression	Social worker
	Diarrhea	Nurse
	Eating, chewing, or swallowing difficulties	Clinical nutritionist, nurse
	Fatigue	Nurse practitioner or physician extender
	Fever	Nurse
	Mobility or physical issues	Nurse
	Mouth sores	Nurse
	Nausea or vomiting	Nurse
	Numbness	Nurse
	Pain	Physician
	Sexual issues	Nurse
	Skin rash	Nurse
	Sleep issues	Nurse practitioner or physician extender
	Swelling	Nurse
	Weight change	Clinical nutritionist, nurse

#### Prototype Design and Development

##### User-Centered Design

The PatientCareAnywhere prototype was developed using an iterative user-centered design approach, in which targeted end users (patients with cancer) and other key stakeholders (eg, caregivers and health care professionals) were involved in the design and development process to ensure the intervention aligns with the needs and preferences of patients newly diagnosed with cancer (target population). Research has shown that involving stakeholders throughout intervention development and evaluation is essential to increasing user acceptance and intervention effectiveness [46,47]. In addition, an expert panel with expertise in the fields of supportive care, oncology, nursing, mental health, outcome research, palliative care, and patient education was assembled to provide continuous guidance and consultation on our prototype design and evaluation efforts.

##### Stakeholder Input

Feedback from patients with cancer and caregivers was largely unanimous in agreement with the idea of a system such as PatientCareAnywhere and the functions that they would like to see implemented. Notably, they all expressed strong support for social networking functions, the ability to keep track of appointments and medical records, access to tailored recommendations for educational support materials and local events and support groups, and the ability to report symptoms at any time that would send alerts to their care team. Patients expressed a strong interest in being able to connect with other patients with cancer who are going through or have been through the same experiences. Caregivers expressed support for the ability to connect with other caregivers to build a support network of others who are also going through the same caregiver experiences. Finally, patients and caregivers felt that the ability to create “help requests” that they could share with their network would make the logistics involved with having cancer and caring for someone with cancer a lot easier. All participants felt that they would like to use PatientCareAnywhere when it was available and that it would be a great resource for others in the same position. The only barriers that these focus groups identified involved possibly leaving out those who are not as technology savvy. However, each group concluded that most people have someone around who is able to help them with the technology.

Feedback from the expert panel highlighted a number of features that they wanted to see implemented in PatientCareAnywhere and the barriers that they foresaw in using PatientCareAnywhere. Overall, the expert panel liked the idea of a system such as PatientCareAnywhere for clinic use and clinic-based research. All members of the expert panel immediately recognized the benefits of having features such as social networking, tailored educational materials, event recommendations, and symptom reporting and management for patients with cancer and felt that PatientCareAnywhere would enable them to provide better care to their patients. The expert panel members also wanted to have the information from PatientCareAnywhere to be integrated into the EHR or have the 2 systems “speak” to each other so that they only had to enter information into 1 system, and it would automatically populate into both systems. The members also wanted to have additional clinical research features available as part of the initial biopsychosocial screening tool to deliver specialized questionnaires to the patients who are part of different research studies at City of Hope.

### Ethical Considerations

All study procedures and assessments were reviewed and approved by the City of Hope Institutional Review Board before participant enrollment (institutional review board #15025). Written informed consent was provided by all study participants recruited for the usability testing (phase 1) and pilot RCT (phase 2), and all participants were provided the ability to opt out of the study at any time. To ensure participant privacy and confidentiality, study data were deidentified using participant ID numbers. The mapping between participant IDs and actual participant identities was maintained by the City of Hope research team in a password-protected electronic file. Each study participant was given a unique participant login ID to access the prototype system, which also enabled researchers to retrieve information related to a specific participant. Usability testing participants (phase 1) were compensated US $50 for their time. Pilot RCT participants (phase 2) received a US $100 stipend as compensation for the time spent in the study.

### Phase 1: Usability Testing

#### Overview

We conducted 2 types of usability testing to evaluate the usability, usefulness, and acceptability of the prototype system. The first usability test was “design oriented” and conducted after wireframes (schematics showing information elements and page flows) were produced. This allowed us to resolve initial design issues before significant development efforts took place. Once most of the development work was completed, we then conducted “metric-oriented” usability tests to formally evaluate the usability of the PatientCareAnywhere using quantitative assessments.

#### Study Participants and Design

To be eligible to participate in usability testing, patients were required to be (1) aged ≥21 years, (2) diagnosed with any cancer, (3) currently receiving any type of cancer treatment, (4) treated on an outpatient status (participation was suspended during hospitalization), (5) fluent in English, and (6) able to access the internet at home. Caregivers, friends, and family members of patients with cancer were also eligible to participate in the study. Those with evidence of cognitive or psychological impairment as well as prisoners and pregnant women were ineligible. Participants were also excluded if they were currently participating in another psychosocial study.

All patients with cancer and caregivers were recruited from City of Hope, a National Cancer Institute–designated comprehensive cancer center in Duarte, California, via physician referrals, subject recruitment flyers, and a touch screen biopsychosocial screening system (SupportScreen [45]), which included a question about participating in this study. Trained research assistants approached potentially eligible patients and discussed study participation either in person during an already scheduled clinic visit or via telephone. Interested patients were then screened for eligibility criteria, and those eligible wishing to enroll provided written informed consent. All participants consented before study participation and were enrolled between March and April 2016.

Each participant completed a 60-minute one-on-one usability testing session, in which they completed specific tasks using the prototype, and an observer recorded how the tasks were completed (or failed). Participants were asked to talk aloud as they performed the tasks. After completing all assigned tasks, participants for the second usability test also completed self-report measures to evaluate perceived usability, usefulness, and acceptability of the PatientCareAnywhere prototype.

Usability testing sessions were audio recorded using encrypted audio recorders and professionally transcribed. The audio files were transmitted via secure protocols to an encrypted project folder on a secure file server at City of Hope. The original audio files were permanently deleted from the audio recorders once uploaded to the file server.

#### Measures

##### Usability

The System Usability Scale (SUS) is a validated and widely used 10-item usability measure [48]. Participants’ scores for each item are added together and then multiplied by 2.5 to convert the original scores of 0 to 40 to 0 to 100, with higher scores indicating higher usability [48]. Overall SUS scores ≥70.0 are considered above average in terms of acceptable usability [49,50].

###### Usefulness

Participants also completed a 35-item Usefulness Questionnaire, which was developed specifically for PatientCareAnywhere and includes statements assessing the usefulness and design features of the system. Participants rated their level of agreement with each statement using a 5-point Likert scale ranging from 1=“strongly disagree” to 5=“strongly agree.”

#### Data Analytic Plan

Data were collected and analyzed using SPSS (IBM Corp). Descriptive statistics (eg, means, frequencies, percentages) were used to characterize the sociodemographic and clinical characteristics of the study sample. Summary statistics were used to describe the usability outcomes, including overall SUS scores and perceived acceptability and usefulness ratings.

### Phase 2: Pilot RCT

#### Study Participants and Design

Textbox 1 shows the inclusion and exclusion criteria for the pilot RCT.

**Inclusion and exclusion criteria.**

**Inclusion criteria**

- At least 21 years of age
- Diagnosis of breast, lung, or prostate cancer at any stage
- Currently being treated on an outpatient basis
- Life expectancy of at least 6 months
- Fluent in English
- Have home internet access

**Exclusion criteria**

- Clinical evidence of cognitive or psychological impairment
- Prisoners and pregnant women
- Currently participating in other psychosocial studies

Study recruitment included physician referrals, advertisements and flyers, and a patient health care portal (SupportScreen [45]) from City of Hope. Participants were enrolled between October 2017 and September 2019. All study participants were screened for complete eligibility criteria and provided written informed consent before study participation. Consented participants were randomized to either the PatientCareAnywhere intervention or usual care control condition using a computer-based random assignment program using a 1 to 1 ratio. Due to the nature of the study, it was not possible to blind participants’ study conditions. Participants in both the intervention and control groups participated in their respective study arm for a 3-month period and completed a baseline assessment at the time of enrollment (T1), which included a sociodemographic questionnaire and 2 biopsychosocial questionnaires assessing HRQOL, as measured by the Functional Assessment of Cancer Therapy-General (FACT-G) [51], and cancer-related self-efficacy, as measured by the Self-Efficacy for Managing Chronic Disease (SEMCD) [52]. Follow-up assessments (FACT-G and SEMCD) were completed monthly until the end of participation, resulting in 3 additional time points: 4 weeks from baseline (T2), 8 weeks from baseline (T3), and 12 weeks from baseline (T4). Table 2 outlines the procedures conducted at different time points of the RCT.

**Table 2:** Procedures conducted at different phases of the pilot randomized controlled trial.

Time	Patient tasks	Clinical team tasks
Enrollment	All: complete sociodemographic questionnaire at clinic All: complete baseline FACT-G^a^ and SEMCD^b^ (T1^c^) All: set up an account for and receive an orientation to the PatientCareAnywhere system	All: enter clinic data into trial management system
Once a week	Intervention: use the PatientCareAnywhere system, including the symptom reporting feature	None
Every month	All: complete FACT-G and SEMCD after 1 month (T2^d^) and 2 months (T3^e^) of participation	All: enter survey data collected on paper into the system
Before every visit	None	Intervention: ensure symptom assessment report from PatientCareAnywhere is either printed or available on computer
During every visit	Intervention: review symptom assessment report with the provider	Intervention: review symptom assessment report with the patient
Conclusion of participation	All: complete FACT-G and SEMCD after 3 months of participation (end of the study; T4^f^)	All: enter all paper-based data into trial management system Intervention: compile metrics of PatientCareAnywhere system use (eg, frequency of use and time spent)

^a^FACT-G: Functional Assessment of Cancer Therapy-General.

^b^SEMCD: Self-Efficacy for Managing Chronic Disease.

^c^At the time of enrollment (baseline).

^d^4 weeks after baseline (first midpoint of the study).

^e^8 weeks after baseline (second midpoint of the study).

^f^12 weeks after baseline (end of participation).

#### Study Conditions

##### Intervention Condition

Patients in the intervention group were encouraged to use PatientCareAnywhere at least weekly to not only report symptoms when necessary but also use other features of the site, such as the education content. Reminder emails were sent to patients to encourage the use of the system after 1 week of inactivity. Patient-reported symptoms of moderate or worse severities triggered email alerts to the study coordinators for triage, who then contacted appropriate providers or supportive care staff to address the patient’s concerns.

##### Control Condition

Patients in the control group received usual care, including a 1-time use of SupportScreen for symptom checking at the clinic during initial treatment consultation after a cancer diagnosis. The use of SupportScreen could also trigger the delivery of consultation, print patient education materials, and specialist referrals.

#### Measures

##### Sociodemographic and Cancer-Specific Characteristics

At baseline, before the intervention, patients self-reported sociodemographic information (eg, age, race, ethnicity, education, and income) and clinical information (eg, cancer diagnosis and stage of cancer), which were confirmed via medical record review.

##### Health-Related Quality of Life

The FACT-G is a 27-item self-report questionnaire designed to measure 4 domains of HRQOL in patients with cancer, including emotional, functional, physical, and social well-being [51]. Patients rate the degree to which the items applied to them over the past 7 days using a 5-point response scale ranging from 1=“not at all” to 5=“very much.” Total FACT-G scores range from 0 to 108, with a higher score indicating better quality of life.

##### Patient Self-Efficacy

Patient self-efficacy is an essential component of the treatment and management of illnesses, including cancer. The 6-item SEMCD scale measures patients’ confidence in their ability to manage fatigue, physical discomfort or pain, emotional distress, and other symptoms or health problems; to carry out different tasks or activities to reduce the need to see a physician; and to do things other than taking medication to reduce illness effects [52,53]. Items are rated on a 10-point scale ranging from 1=“not at all confident” to 10=“totally confident,” and scores are averaged across items. The final score (mean of the 6 items) ranges from 0 to 10, with higher scores indicating greater self-efficacy.

##### Intervention Use

PatientCareAnywhere tracked the frequency with which participants accessed the intervention over the 3-month study period. The system also recorded participants’ responses to multiple symptom assessments and the time (minutes) it took to complete each assessment.

#### Sample Size

The primary goal of the pilot RCT was to compare the FACT-G change across time in the intervention group with the FACT-G change across time in the control group. The sample size calculation was based on prior research that established the minimally important difference for the total FACT-G ranges from 4 to 7 points [54-56]. Specifically, a sample size of 72 participants (36 participants per group) would achieve >80% power to detect a difference in mean changes of 7 (with SD of 12 at both time points and a correlation between measurement pairs of 0.65). The significance level is .05 using a 2-sided, 2-sample *t* test.

#### Data Analytic Plan

Descriptive statistics (eg, means, frequencies, and percentages) were used to characterize the sociodemographic and disease characteristics of the RCT participants. Demographic differences between intervention and control groups were evaluated using *t* test for continuous variables and Fisher exact test for categorical variables. Regarding FACT-G and SEMCD scores, independent sample *t* test was used to compare mean differences (ie, mean difference between pre- and postintervention scores) between the 2 groups at T4. All statistical analyses were conducted using SPSS.

#### Hypotheses

We hypothesized that at postintervention, patients randomized to the PatientCareAnywhere intervention would report better HRQOL outcomes, as measured by the FACT-G (primary hypothesis), and self-efficacy, as measured by the SEMCD (secondary hypothesis), compared with patients randomized to the usual care control condition.

## Results

### Phase 1: Usability Testing

#### Participant Characteristics

A total of 11 participants (patients: n=4 and caregivers: n=7) participated in usability testing with a prototype of the PatientCareAnywhere system. This sample size was justified based on previous usability research demonstrating that 5 to 7 participants is sufficient to reveal about 80% of the usability issues [57]. Table 3 presents the sociodemographic characteristics of the usability testing sample. Patients were mostly non-Hispanic (7/11, 64%) and White (10/11, 91%), with an average age of 50 (SD 6.8) years. The average age of caregivers was 44 (SD 20) years.

**Table 3:** Sample characteristics of usability testing participants (N=11).

			Patients (n=4)		Caregivers (n=7)
Age (y), mean (SD; range)			50.3 (6.8; 44-59)		43.7 (20.4; 23-73)
**Gender, n (%)**					
	Man	0 (0)		4 (57.14)	
	Woman	4 (100)		3 (42.86)	
**Race, n (%)**					
	Asian	1 (25)		0 (0)	
	White	3 (75)		7 (100)	
**Ethnicity, n (%)**					
	Hispanic or Latino	2 (50)		1 (14.29)	
	Non-Hispanic	2 (50)		5 (71.43)	
	Unknown	0 (0)		1 (14.29)	

#### Usability Outcomes

##### Qualitative Results

Individual interviews with patients with cancer and caregivers were conducted to evaluate the usability and usefulness of the PatientCareAnywhere system. The interviews consisted of asking the participants to complete a list of tasks that addressed each of the features of the PatientCareAnywhere system (eg, where to find certain information on the page or how to complete a symptom report) and recording the time it took for each task to be completed as well as identifying any tasks that were difficult to complete. Multimedia Appendix 3 provides the list of tasks that were asked of participants. In addition to the task-completion activity, we solicited feedback from the participants on the site functions, features, and design as well as their ideas for improvement. All participants were able to complete the tasks within 5 seconds of being asked, and no participant experienced confusion about navigating the site and completing specific activities.

Participants also had high levels of satisfaction with the PatientCareAnywhere design, features, and functionality of the system. Specifically, patients enjoyed the ability to connect with friends, family, community organizations, other patients with cancer and survivors of cancer, and their care team. They felt that the PatientCareAnywhere layout made sense to them as users, and they did not find any parts of the pages to be confusing. The patients uniformly liked the layout of the biopsychosocial screening tool and preferred the idea that they only had to give responses to the topics that they were concerned about on the tool. They also felt that the personalized recommendations would be a great asset to them during their cancer journey and were especially happy about being able to report their symptoms at any time. In addition, patients really liked the “one-stop-shop” idea of PatientCareAnywhere—the ability to keep track of their appointments and medical information in the same place as connecting with friends and family and finding local events and support groups. All patients felt that the wireframes were well thought out, and each one asked when the system would be available for use at City of Hope.

The caregivers also highly praised the PatientCareAnywhere wireframes. All caregiver participants felt that the wireframes were laid out in a logical manner. They particularly liked having access to their loved one’s medical records and appointments (given only with the caregiver permission level), and they felt that this system would make caregiving a much easier experience. Other features that the caregivers highlighted would make a difference for them were the ability to complete a symptom report for their loved one (patient) and the “help request” feature that would allow caregivers (and patients) to send requests for help (eg, assistance with transportation) to their PatientCareAnywhere friends. The PatientCareAnywhere friends can respond to the email request if they can help and this affirmation is noted by the PatientCareAnywhere system. Overall, patients and caregivers did not have any trouble identifying how to complete the biopsychosocial screening tool and where to find recommendations, medical information, educational materials, and local events on the PatientCareAnywhere wireframes.

##### Quantitative Results

The average SUS total score was 84.09 (SD 10.02; range 75.00-100.00), which was well above the predetermined 70-point threshold reflecting “excellent” usability (Table 4). Regarding the Usefulness Questionnaire, participants agreed or strongly agreed with all 35 statements (refer to Tables 5 and 6, which include average ratings for each statement). Specifically, patients and caregivers rated the usefulness of PatientCareAnywhere site features from 4.00 to 5.00 (Table 5) or 3.86 to 4.86 (Table 6) and, respectively (4=“agree” and 5=“strongly agree”).

**Table 4:** Results from the System Usability Scale questionnaire (N=11)^a^.

Item	Score, mean (SD)
1. I think that I would like to use this system frequently.	4.18 (0.60)
2. I found the system unnecessarily complex.	1.64 (0.82)
3. I thought the system was easy to use.	4.36 (0.48)
4. I think that I would need the support of a technical person to be able to use this system.	1.36 (0.70)
5. I found the various functions in this system were well integrated.	4.36 (0.48)
6. I thought there was too much inconsistency in this system.	1.55 (0.70)
7. I would imagine that most people would learn to use this system very quickly.	4.27 (0.42)
8. I found the system very cumbersome to use.	1.73 (1.03)
9. I felt very confident using the system.	4.36 (0.67)
10. I needed to learn a lot of things before I could get going with this system.	1.64 (0.67)

^a^Total usability score is a sum of individual items multiplied by 2.5 to convert original scores of 0 to 40 to 0 to 100. Possible item responses range from 1 (strongly disagree) to 5 (strongly agree).

**Table 5:** Results from the Patient’s Usefulness Questionnaire (N=4)^a^.

Item number and item		Score, mean (SD)
**In managing my cancer care, it is useful to...**		
	1. Connect with friends, family, and doctors/nurses through private messaging and wall posting features.	4.75 (0.50)
	2. Receive education recommendations that are tailored to my medical situation and/or personal needs.	5.00 (0.00)
	3. View support group recommendations that are tailored to my needs.	4.25 (0.50)
	4. View recommendations for local classes and events that are tailored to my needs.	4.25 (0.50)
	5. Be able to create help requests that are sent out to caregivers and/or friends.	4.75 (0.50)
	6. Report symptoms via the symptom reporting tool.	5.00 (0.00)
	7. Be able to track my symptoms over time via the symptom reporting tool.	4.25 (0.50)
	8. View the educational articles that were recommended to me based off the reported symptoms.	5.00 (0.00)
	9. Have access to my medication and supplement list.	4.75 (0.50)
	10. Have access to my laboratories and tests results.	5.00 (0.00)
	11. Have access to my other medical records.	5.00 (0.00)
	12. Be able to add additional medical information or upload other medical documents.	4.75 (0.50)
	13. View the care team members that have received referrals regarding my personal or medical needs.	4.75 (0.50)
	14. See the events that are scheduled at the City of Hope.	4.00 (0.82)
	15. See the events for which I am registered.	4.50 (0.58)
	16. Add my own events to my calendar.	4.50 (0.58)
	17. View the medical appointments that are scheduled for me.	5.00 (0.00)
	18. View the help requests that have been sent out.	5.00 (0.00)
	19. See which classes, events and support groups are available at the City of Hope.	4.25 (0.50)
	20. See which classes, events and support groups are available in my local area.	4.25 (0.50)
	21. Be able to register for a class, event or support group.	4.50 (0.58)
	22. Read a description of the class/event/support group and the event leader’s contact info.	4.50 (0.58)
	23. Be able to read the educational content/articles that have been recommended to me.	4.75 (0.50)
	24. Be able to save articles that I want to reference later into a “Favorites” area.	4.75 (0.50)
	25. Be able to browse educational materials by category.	4.50 (0.58)
	26. Be able to request additional information about a topic.	5.00 (0.00)
	27. Be able to share an educational article with the PCA^b^ administrators so that they could add it to PCA.	4.75 (0.50)
**In general, I feel...**		
	1. Comfortable using PCA on my own.	4.50 (0.58)
	2. That PCA is an easy site to navigate.	4.50 (0.58)
	3. That the overall look-and-feel of PCA is appealing.	4.50 (0.58)
	4. That the overall organization of PCA is logical.	5.00 (0.00)
	5. That for noncritical medical situations, I would rather get information and nurse help via PCA instead of having an in-person doctor’s appointment.	4.50 (0.58)
	6. That I would recommend PCA to other caregivers.	4.75 (0.50)
	7. That I would recommend PCA to other patients.	4.75 (0.50)
	8. That cancer centers should use PCA as part of their standard care practices.	4.75 (0.50)
Total score		4.66 (0.28)

^a^The highest score is 5.00 (strongly agree) and the lowest score is 1.00 (strongly disagree).

^b^PCA: PatientCareAnywhere.

**Table 6:** Results from the Caregiver’s Usefulness Questionnaire (N=7)^a^.

		Score, mean (SD)
**In caring for my family member/friend who has cancer, it is useful to...**		
	1. Connect with her/him and others through private messaging and wall posting features.	3.86 (0.90)
	2. Receive education recommendations that are tailored to my friend/family member’s medical situation.	4.86 (0.38)
	3. View support group recommendations that are tailored to my friend/family member’s needs.	4.71 (0.49)
	4. View recommendations for local classes and events that are tailored to my friend/family member’s needs.	4.71 (0.49)
	5. Be able to create help requests that are sent out to other caregivers and/or friends.	4.71 (0.49)
	6. Report symptoms via the symptom reporting tool on behalf of my family member/friend.	4.71 (0.49)
	7. Be able to track her/his symptoms over time via the symptom reporting tool.	4.71 (0.49)
	8. View the educational articles that were recommended for her/him based off the reported symptoms.	4.86 (0.38)
	9. Have access to her/his medication and supplement list.	4.86 (0.38)
	10. Have access to her/his laboratories and tests results.	4.86 (0.38)
	11. Have access to her/his other medical records.	4.86 (0.38)
	12. Be able to add additional medical information or upload other medical documents.	4.86 (0.38)
	13. View the care team members that have received referrals for my family member/friend.	4.71 (0.49)
	14. See the events that are scheduled at the city of hope.	4.43 (0.79)
	15. See the events for which he/she is registered.	4.43 (0.53)
	16. Add my own events to my calendar.	4.29 (0.95)
	17. Add events for my family member/friend.	4.29 (0.95)
	18. View the medical appointments that are scheduled for my family member/friend.	4.86 (0.38)
	19. View the help requests that have been sent out.	4.71 (0.49)
	20. See which classes, events and support groups are available at the city of hope.	4.43 (0.98)
	21. See which classes, events and support groups are available in my local area.	4.43 (0.79)
	22. View the classes, events and support groups that are recommended for my family member/friend.	4.43 (0.79)
	23. Be able to register my family member/friend for a class, event or support group on their behalf.	4.29 (0.76)
	24. Read a description of the class/event/support group and the event leader’s contact info.	4.43 (0.79)
	25. Be be able to read the educational content/articles that have been recommended to my family member/friend.	4.86 (0.38)
	26. Be able to save articles that i want to reference later into a “favorites” area.	4.86 (0.38)
	27. Be able to browse educational materials by category.	4.71 (0.49)
	28. Be able to request additional information about a topic.	4.71 (0.49)
	29. Be able to share an educational article with the PCA^b^ administrators so that they could add it to PCA.	4.43 (0.53)
**For me personally as a caregiver, it is useful to...**		
	1. Have education recommendations that are tailored to my role as caregiver	4.86 (0.38)
	2. Have support group recommendations that are tailored to my role as caregiver.	4.29 (0.76)
	3. Have recommendations for local classes and events that are tailored to my role as caregiver.	4.43 (0.53)
**In general, I feel...**		
	1. Comfortable using PCA on my own.	4.57 (0.79)
	2. That PCA is an easy site to navigate.	4.43 (0.79)
	3. That the overall look-and-feel of PCA is appealing.	4.43 (0.79)
	4. That the overall organization of PCA is logical.	4.57 (0.53)
	5. That for noncritical medical situations, I would rather get information and nurse help via PCA instead of having an in-person doctor’s appointment.	4.29 (0.76)
	6. That I would recommend PCA to other caregivers.	4.71 (0.76)
	7. That I would recommend PCA to other patients.	4.71 (0.76)
	8. That cancer centers should use PCA as part of their standard care practices.	4.57 (0.79)
Total scores		4.59 (0.23)

^a^The highest score is 5.00 (strongly agree) and the lowest score is 1.00 (strongly disagree).

^b^PCA: PatientCareAnywhere.

#### Phase 2: Pilot RCT

##### Participant Characteristics

A total of 72 patients with cancer were enrolled and individually randomized (1:1) to the PatientCareAnywhere intervention (n=36, 50%) or usual care control condition (n=36, 50%) for 3 months. The following analysis was limited to 59 participants who completed at least 2 of the questionnaires (FACT-G and SEMCD): 28 (47%) patients in the intervention group and 31 (53%) patients in the control group. Of note, there were no significant differences in demographic characteristics between the included (59/72, 82%) and excluded (13/72, 18%) participants (Multimedia Appendix 4). Table 7 summarizes the pilot RCT participants’ sociodemographic and cancer-related characteristics, with no significant between-group differences. Overall, the RCT participants had a mean age of 53.85 (SD 12.37) years and were predominantly women (49/59, 83%), White (41/59, 69%), and non-Hispanic or Latino (41/59, 69%). Most participants were married (43/59, 73%), had at least a college degree (33/59, 56%), and earned >US $100,000 (29/59, 49%). The most common diagnosis was breast cancer (43/59, 73%) and nonmetastatic (stages 0-III; 33/59, 56%).

**Table 7:** Sample characteristics of the pilot randomized controlled trial participants (N=59).

		Intervention (n=28)	Control (n=31)	*P* value
Age (y), mean (SD; range)		54.82 (12.32; 34-77)	52.97 (12.37; 30-79)	.57
**Gender, n (%)**				.73
	Man	4 (14.29)	6 (19.35)	
	Woman	24 (85.71)	25 (80.65)	
**Race, n (%)**				.55
	Asian American	6 (21.43)	4 (12.90)	
	Black or African American	2 (7.14)	5 (16.13)	
	White	20 (71.43)	21 (67.74)	
	Unknown	0	1 (3.23)	
**Ethnicity, n (%)**				.24
	Hispanic or Latino	9 (32.14)	6 (19.35)	
	Non-Hispanic	17 (60.71)	24 (77.42)	
	Unknown or not reported	2 (7.14)	1 (3.23)	
**Marital status, n (%)**				.07
	Single	2 (7.14)	6 (19.35)	
	Married	19 (67.86)	24 (77.42)	
	Separated or divorced	5 (17.86)	1 (3.23)	
	Widowed	2 (7.14)	0 (0)	
**Education, n (%)**				.23
	Less than high school	2 (7.14)	1 (3.23)	
	High school	5 (17.86)	7 (22.58)	
	College	13 (46.43)	20 (64.52)	
	Graduate school	8 (28.57)	3 (9.68)	
**Household income (US $), n (%)**				.93
	<20,000	4 (14.29)	4 (12.90)	
	20,000-29,999	2 (7.14)	1 (3.23)	
	30,000-49,999	2 (7.14)	4 (12.90)	
	50,000-69,999	3 (10.71)	5 (16.13)	
	70,000-99,999	3 (10.71)	2 (6.45)	
	>100,000	14 (50)	15 (48.39)	
**Cancer, n (%)**				.57
	Breast	20 (71.43)	23 (74.19)	
	Lung	5 (17.86)	2 (6.45)	
	Prostate	2 (7.14)	3 (9.68)	
	Unknown	1 (3.57)	3 (9.68)	
**Disease stage, n (%)**				.86
	Stage 0	2 (7.14)	3 (9.68)	
	Stage 1	6 (21.43)	5 (16.13)	
	Stage 2	4 (14.29)	7 (22.58)	
	Stage 3	2 (7.14)	4 (12.90)	
	Stage 4	8 (28.57)	7 (22.58)	
	Unknown	6 (21.43)	5 (16.13)	

#### Study Outcomes

##### Health-Related Quality of Life

The mean difference in FACT-G scores between the preintervention (T1; baseline) and postintervention (T4; 12-week postbaseline) assessments of each patient (Table 8) for the intervention group was 6.08 (SD 15.26), indicating an improvement in HRQOL among patients who received PatientCareAnywhere. For the control group, the mean difference in FACT-G scores between the preintervention (T1) and postintervention (T4) assessments was –2.95 (SD 10.63), indicating a worsening of HRQOL among patients who received usual care. The between-group difference was statistically significant (*P*=.01), with a medium effect size (Cohen *d*=0.70).

**Table 8:** Results from the pilot randomized controlled trial.

Group		Mean difference^a^ (SD)	*P* value^b^
**HRQOL^c^ (FACT-G^d^)**			
	Control (n=31)	−2.95 (10.63)	*.01*
	Intervention (n=26)	6.08 (15.26)	*.01*
**Self-efficacy (SEMCD^e^)**			
	Control (n=31)	−0.84 (11.20)	.09
	Intervention (n=27)	4.22 (10.91)	.09

^a^Mean difference between preintervention (T1; baseline) and postintervention (T4; 12 weeks from baseline) scores.

^b^Significant *P* values (*P*<.05) are italicized.

^c^HRQOL: health-related quality of life.

^d^FACT-G: Functional Assessment of Cancer Therapy-General.

^e^SEMCD: Self-Efficacy for Managing Chronic Disease.

##### Patient Self-Efficacy

Similarly, the mean difference in SEMCD scores between the preintervention (T1; baseline) and postintervention (T4; 12-week postbaseline) assessments (Table 8) for the intervention group was 4.22 (SD 10.91) and for the control group was –0.84 (SD 11.20). However, the between-group difference was not statistically significant (*P*=.09), with a small-to-medium effect size (Cohen *d*=0.46).

##### Intervention Use

Overall, 61% (17/28) of the patients in the intervention group were classified as “Frequent Users,” defined as having accessed the PatientCareAnywhere site at least 5 times during the study. Among the frequent users, the mean difference between the first and last FACT-G scores was 7.12 (SD 15.4), which was statistically significantly higher than that of the control group (*P*=.007), with a large effect size (Cohen *d*=0.80). The mean difference between the first and last SEMCD scores (mean 5.47, SD 6.43) was also statistically significantly better than that of the control group (*P*=.03), with a medium effect size (Cohen *d*=0.71). In comparison, among the infrequent users (n=11), the mean difference between the first and last scores on the FACT-G (mean 4.02, SD 17.39; *P*=.10) and SEMCD (mean 0.73, SD 15.37; *P*=.68) did not significantly differ from the control group.

##### Symptom Reporting

Finally, a total of 140 symptom reports were recorded. On average, each symptom reporting session included 4.4 symptoms and lasted for 3.4 minutes.

## Discussion

The primary aim of this study was to evaluate the usability and preliminary efficacy of PatientCareAnywhere, a patient-centered adaptive supportive care system, to improve patient-reported outcomes for patients newly diagnosed with cancer.

### Principal Findings

Both qualitative and quantitative usability testing results were notably positive and support the usability of PatientCareAnywhere. Overall, patients with cancer and caregivers were highly satisfied with the purpose and functions of the intervention, found the content relevant and useful, and expressed strong support for the biopsychosocial screening tool and personalized recommendations. On the basis of participant feedback, several changes were made to the design features: a “help request” function was added, caregivers were given greater access to the patient’s medical information, and symptom reporting was added to the caregiver portal, allowing caregivers to report symptoms on the patient’s behalf. In addition, the quantitative feedback demonstrated a high usability level for PatientCareAnywhere, with an average SUS score of 84.09 (SD 10.02), indicating above-average acceptable usability [49,50]. The scores on the Usefulness Questionnaire also reflected the positive experience that users had with the system and underlined the beliefs of participants that the features of PatientCareAnywhere were acceptable and useful during the cancer care journey.

Results from the pilot RCT demonstrate the preliminary efficacy of the PatientCareAnywhere intervention. Compared with usual care, patients with cancer who received PatientCareAnywhere showed statistically significant improvements in HRQOL from pre- to postintervention scores. While self-efficacy scores also increased in the intervention group, the difference was not statistically significant compared with the control group. When evaluating intervention use, “frequent users” (ie, patients who accessed the intervention at least 5 times during the study) reported greater improvements in both HRQOL and self-efficacy outcomes (medium to large effect sizes) compared with the control group. These results confirmed our hypotheses that routine use of PatientCareAnywhere could result in improved HRQOL outcomes and greater patient self-efficacy among patients newly diagnosed with cancer, and that these effects were more prominent with greater intervention use. Furthermore, patients on average reported about 4 symptoms and completed the symptom assessment in <4 minutes. Notably, this is drastically shorter than SupportScreen, which takes approximately 15 to 20 minutes to complete [45]. This observation indicates that PatientCareAnywhere is also an efficient symptom reporting tool.

There is growing evidence of technology-assisted assessments and interventions enhancing the delivery of patient-centered care through improved symptom monitoring, communication between patients and providers, tailored resources, and patient empowerment and engagement across the continuum of care [42]. Our findings are in line with previous studies that have demonstrated the effectiveness of technology-based interventions in cancer care [42]. Recently, a comprehensive scoping review was conducted on 134 literature reviews of digital health and telehealth interventions across the cancer continuum, in which a majority focused on patients with cancer (n=128) in the active treatment (n=48) and survivorship (n=29) phases using eHealth programs, synchronous telehealth, mobile apps, asynchronous messaging (eg, email), and SMS text messaging [58]. A total of 29 reviews included a meta-analysis, with results signifying positive effects of digital health and telehealth in cancer care on quality of life, psychological outcomes (eg, anxiety and depression), and cancer screenings [58]. Of note, the benefits of digital supportive cancer care interventions have been demonstrated independent of demographic and disease factors [44,59]. The lack of a positive effect on self-efficacy warrants further evaluation. Similar to our findings, an RCT evaluating an internet-based interactive health communication application that allows patients with cancer to monitor their symptoms and provides tailored self-management support reported no significant between-group differences in depression, HRQOL, self-efficacy, and social support, although self-efficacy and HRQOL outcomes significantly worsened over time in the control group [60]. Conversely, an earlier review and meta-analysis of eHealth-based self‐management interventions demonstrated a statistically significant effect on self-efficacy but noted that the effect size was small (<0.4) [61]. A larger sample size may be needed to observe meaningful changes in self-efficacy.

### Strengths and Limitations of PatientCareAnywhere

PatientCareAnywhere was developed using a user-centered design approach to ensure the needs and preferences of patients newly diagnosed with cancer were addressed. Applying user-centered design principles to the overall development of PatientCareAnywhere resulted in a user-friendly, functional, useful, and acceptable supportive care intervention. In addition, the web-based delivery and responsive-design technologies allow patients to access the intervention at anytime and anywhere, including outside of clinic and at home, and use PatientCareAnywhere on multiple devices (eg, desktops, smartphones, and tablets), thereby providing more flexible intervention delivery and reducing common practical barriers to care (eg, transportation issues and scheduling conflicts). Furthermore, by remotely and routinely monitoring patients’ biopsychosocial symptoms and distress, PatientCareAnywhere provides supportive cancer care tailored to their needs. Compared with other distress management systems, additional advantages of PatientCareAnywhere include (1) PatientCareAnywhere has access to numerous validated questionnaires, allowing an institution to pick and choose the ones that are most suitable for their patients and providers; (2) PatientCareAnywhere allows the invocation of another questionnaire for follow-up questions based on the results from a top-level screening tool; (3) PatientCareAnywhere provides a communication platform to allow caregivers, family members, and friends to interact directly with the patient via the system; (4) PatientCareAnywhere allows community organizations to post events and respond to patient requests for help; (5) PatientCareAnywhere delivers tailored and responsive patient education contents that evolve with the patient based on their current needs and concerns; and (6) PatientCareAnywhere is backed by City of Hope’s comprehensive supportive care training program.

This study has some limitations. First, the objectives of this study were to establish the usability and preliminary effects of PatientCareAnywhere rather than investigate intervention efficacy. However, results from this pilot study will inform the next phase of research to conduct a full-scale RCT evaluating the efficacy of PatientCareAnywhere to improve HRQOL and reduce symptom burden compared with usual care. Second, the study was limited to patients diagnosed with breast, lung, or prostate cancer. We initially focused on these 3 common cancers to develop cancer-specific educational materials, with plans to expand to all cancer types (eg, gastrointestinal, gynecologic, head and neck, and hematologic) and treatment options following successful initial pilot testing results. Further study is warranted to adapt PatientCareAnywhere to other types of cancer and examine the extent to which our initial findings are generalizable to patients with different cancer diagnoses. Third, while sufficient for the purposes of our study, sample sizes for usability testing (N=11) and pilot RCT (N=78) were relatively small, limiting power and study results. It is possible that with a larger sample, stronger intervention effects may emerge. Fourth, study participants were limited to patients receiving care at City of Hope and may not be representative of the general cancer population in the United States. Furthermore, the intervention was only available in English and required patients to have internet access to participate in the study, which also may limit the generalizability of our findings. Future studies should investigate the efficacy of PatientCareAnywhere with a more diverse and larger sample of patients with cancer over a longer study period and explore the optimal intervention use to improve patient outcomes.

### Conclusions

Engaging key stakeholders throughout the design and development process helped ensure PatientCareAnywhere was a patient-centered, user-friendly, efficient, and effective supportive care system. Overall, the results from initial pilot testing demonstrate the usability and preliminary efficacy of PatientCareAnywhere to improve HRQOL outcomes among patients newly diagnosed with cancer.

## Appendix

Multimedia Appendix 1 Main components of PatientCareAnywhere.

Multimedia Appendix 2 Screenshots of PatientCareAnywhere.

Multimedia Appendix 3 PatientCareAnywhere usability tasks (phase 1).

Multimedia Appendix 4 Sample characteristics of the included versus excluded pilot randomized controlled trial participants (phase 2).

Multimedia Appendix 5 CONSORT eHEALTH checklist (V 1.6.1).

## Abbreviations

EHR: electronic health record
FACT-G: Functional Assessment of Cancer Therapy-General
HRQOL: health-related quality of life
NCCN: National Comprehensive Cancer Network
RCT: randomized controlled trial
SEMCD: Self-Efficacy for Managing Chronic Disease
SUS: System Usability Scale

## Data Availability

The data sets generated during and analyzed during this study are available from the corresponding author on reasonable request.
