# Supplementary material for: Usability and Preliminary Efficacy of an Adaptive Supportive Care System for Patients With Cancer: Pilot Randomized Controlled Trial
Source: JMIR Cancer. 2024 Jul 10;10:e49703. doi: 10.2196/49703 (PMC11269963; doi:10.2196/49703)
Supplement: Multimedia Appendix 1 [file cancer_v10i1e49703_app1.docx]

Multimedia Appendix 1. Main Components of *PatientCareAnywhere*

| **Components** | **Description** |
| --- | --- |
| **Dashboard** | The home page includes social media support within the patient’s “care circles” (e.g., private messaging or wall posting feature) |
| **My Medical** | This section includes information from the patient’s medical records (e.g., medications, labs/tests, appointments), care team referrals, and messages to care team members. |
| **Report Concerns** | The symptom reporting and management feature allows patients to report their concerns remotely via electronic patient-reported outcome (ePRO) assessments and receive adaptive content based on their current needs. The system automatically notifies the care team about moderate to severe symptom reports via an email/pager alert. Screening summaries and visual report of symptoms over time (line graphs) are also available under “Symptom History." |
| **Help Requests** | Patients/caregivers can post requests for help with specific tasks (e.g., asking for a ride, preparing meals), which their friends and family can sign up for and assist. |
| **My Calendar** | Patients can view their own appointments, create new calendar events, or add events from City of Hope’s calendar (list of cancer-related classes, events, and support groups) to their personal calendar. |
| **Learning Center** | This section includes recommended articles based on the patient’s screening results and self-reported needs (e.g., “*Sexuality and Reproductive Issues”*), as well as access to all available patient educational materials, which are organized by the following content areas: *About Cancer*, *Caregivers*, *Nutrition*, *Questions to Ask Your Doctor,* *Social and Practical Needs*, *Symptom Management*, and *Treatments*. |
| **My Favorites** | Articles of interest from the Learning Center section can be bookmarked and saved under “My Favorites” for easy reference. |
| **Classes & Events, and Support Groups** | These sections provide a comprehensive list of City of Hope’s classes, workshops, and support groups for patients and their families, as well as local cancer-related events and community resources and organizations for individuals impacted by cancer. Patients receive personalized recommendations for City of Hope and community supportive care programs (e.g., gentle restorative yoga, art therapy). |
