# Supplementary material for: Usability and Preliminary Efficacy of an Adaptive Supportive Care System for Patients With Cancer: Pilot Randomized Controlled Trial
Source: JMIR Cancer. 2024 Jul 10;10:e49703. doi: 10.2196/49703 (PMC11269963; doi:10.2196/49703)

Multimedia Appendix 2. Screenshots of *PatientCareAnywhere*

1.
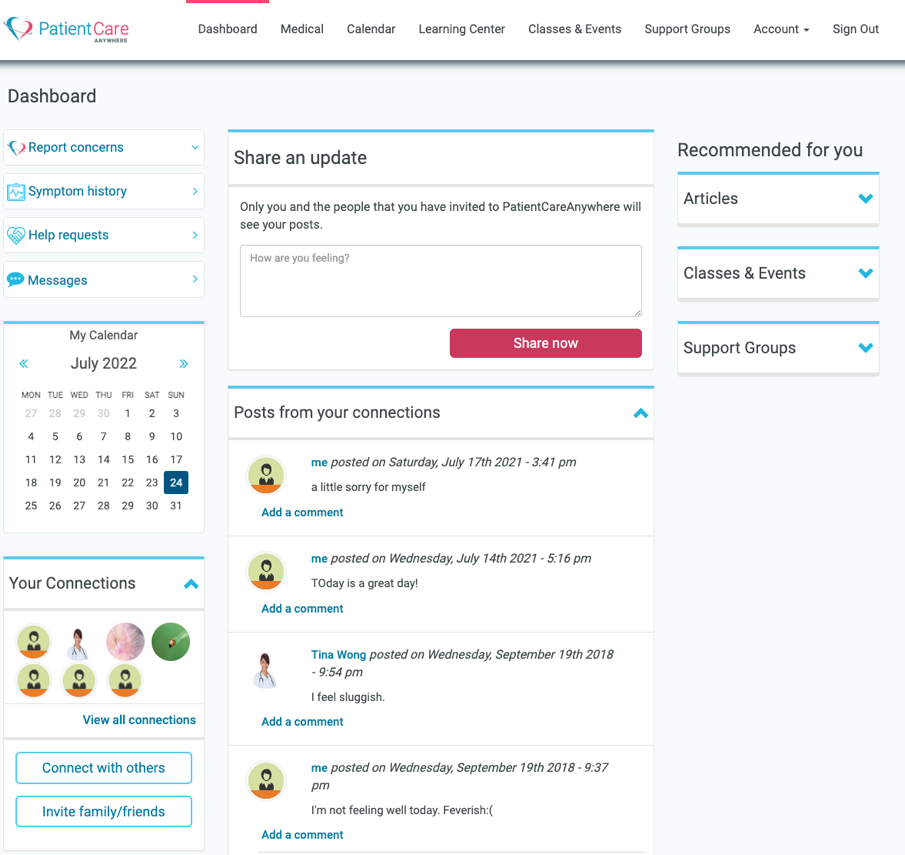
Homepage:
2.
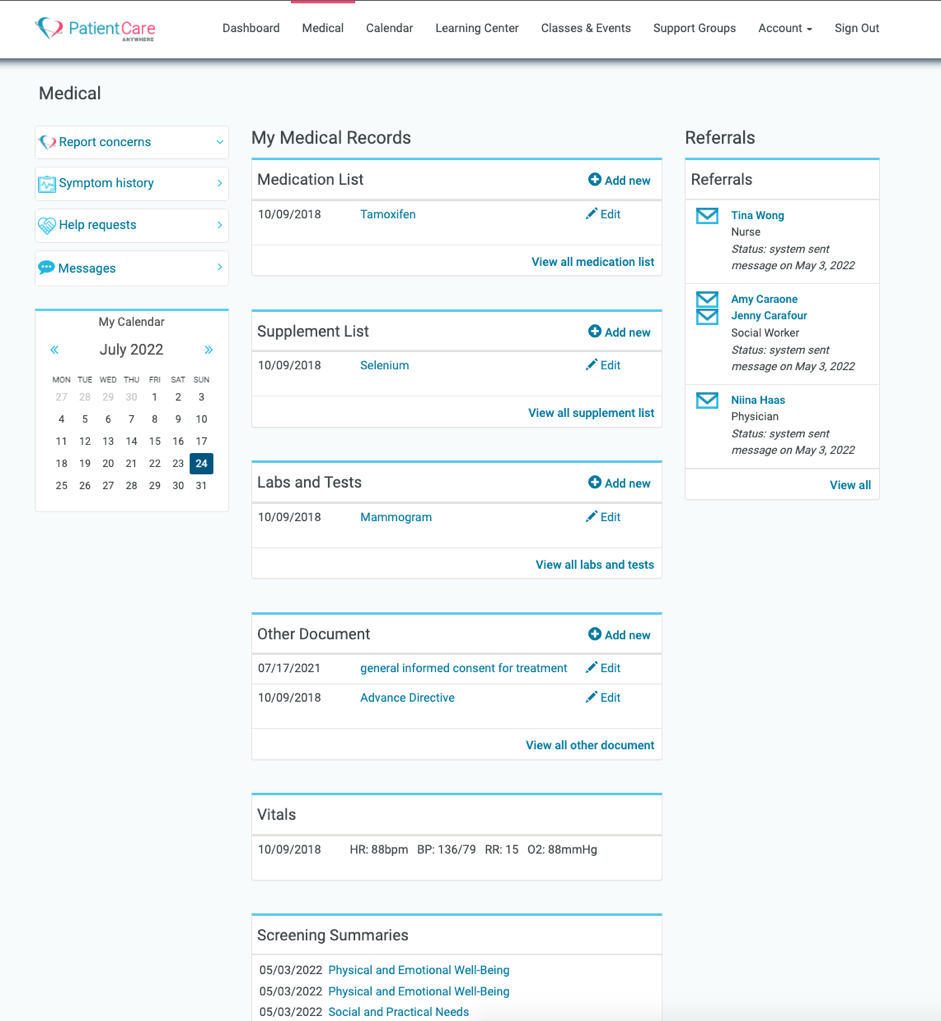
Medical Dashboard:
   1. “Report Concerns” Under Medical:

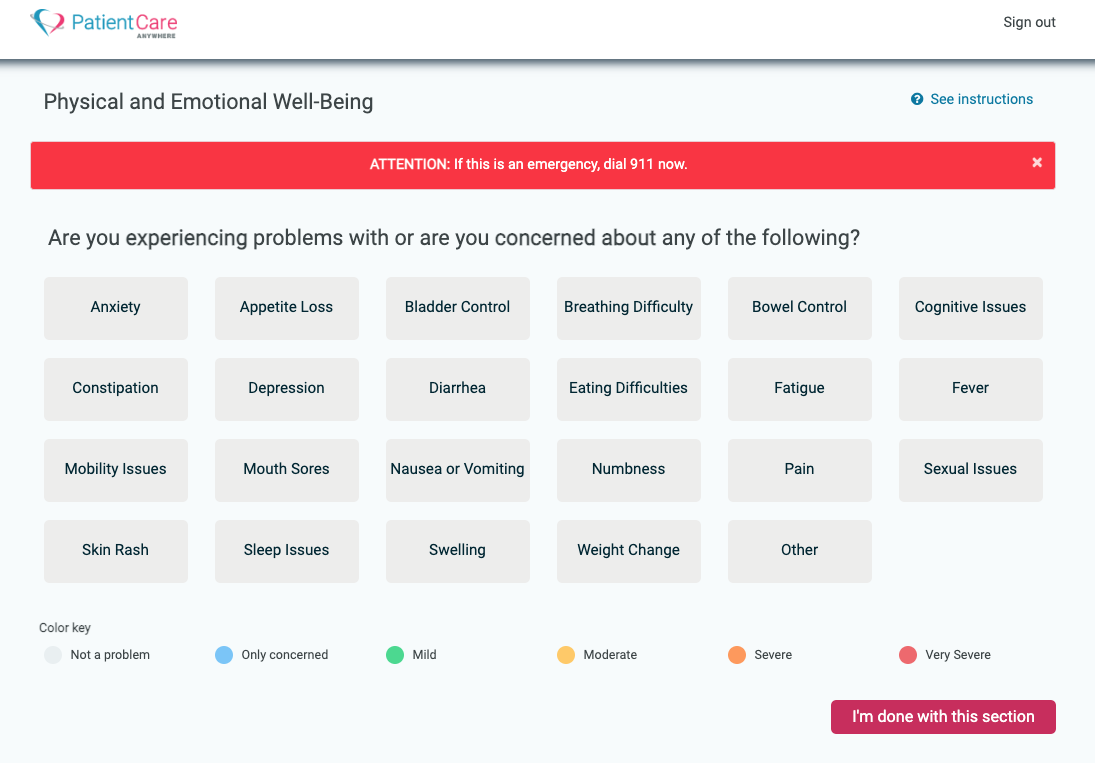

   2. “Symptom History” under Medical:

1. Learning Center:


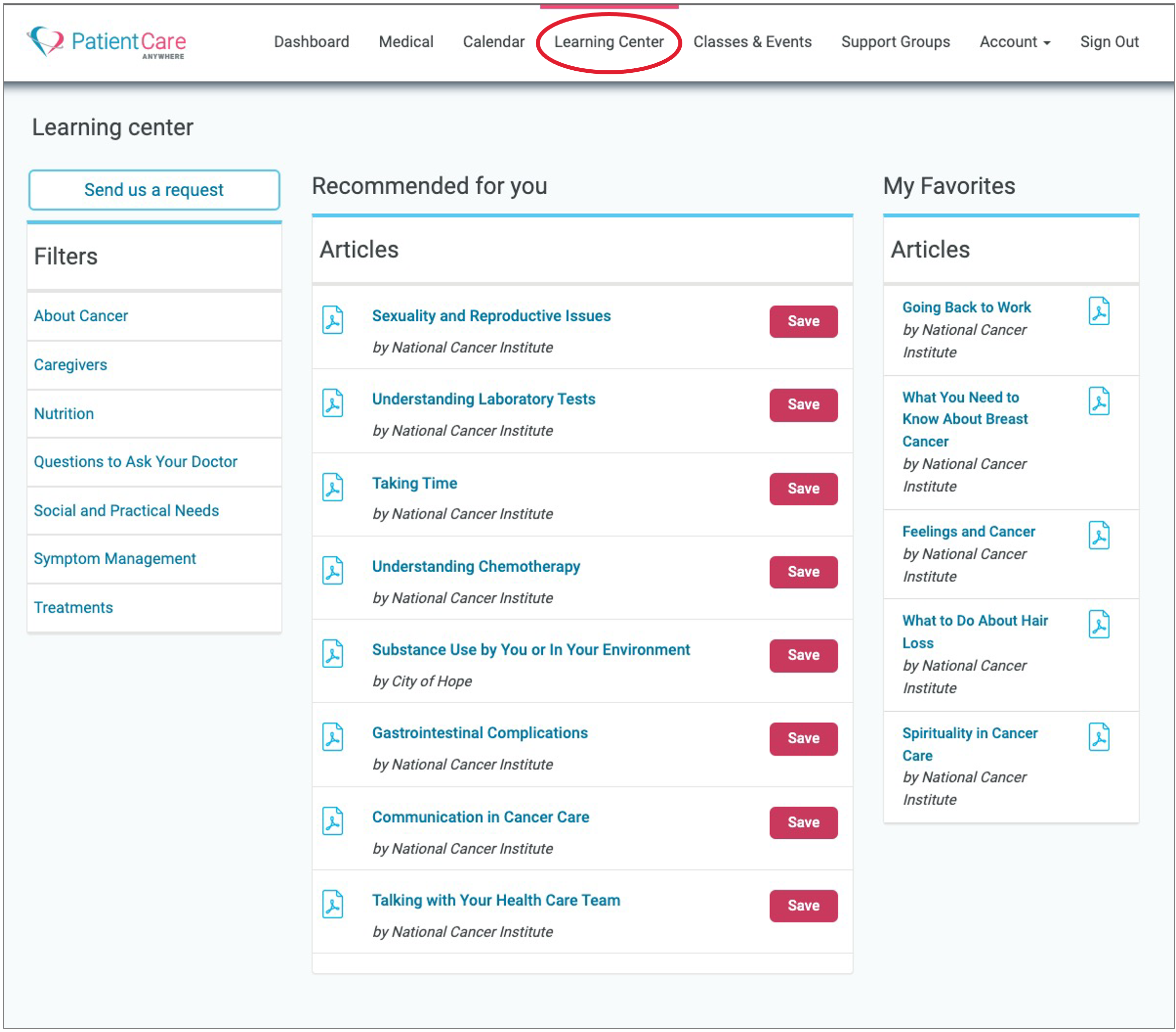


1.
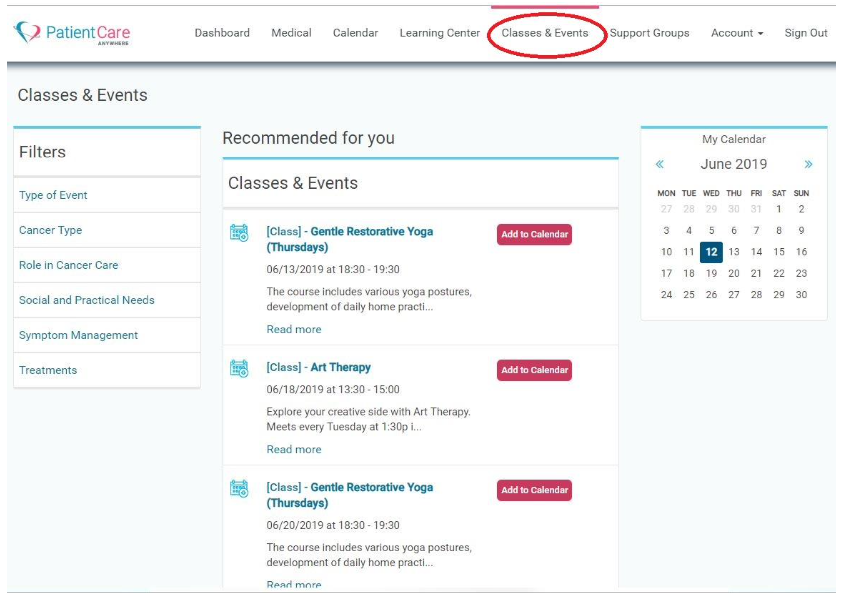
Classes & Events
2. Support Groups


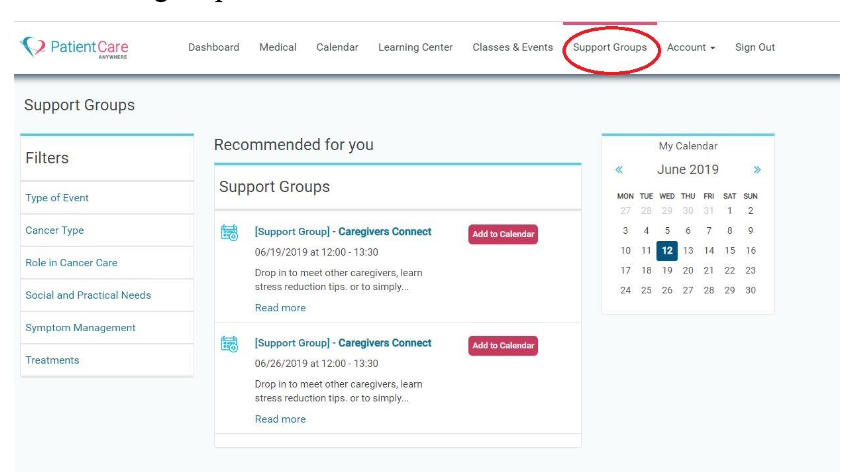

Supplement: Multimedia Appendix 2 [file cancer_v10i1e49703_app2.docx]
