# Supplementary material for: Usability and Preliminary Efficacy of an Adaptive Supportive Care System for Patients With Cancer: Pilot Randomized Controlled Trial
Source: JMIR Cancer. 2024 Jul 10;10:e49703. doi: 10.2196/49703 (PMC11269963; doi:10.2196/49703)
Supplement: Multimedia Appendix 3 [file cancer_v10i1e49703_app3.docx]

Multimedia Appendix 3. *PatientCareAnywhere* Usability Tasks (Phase I)

| ***PatientCareAnywhere* Usability Tasks** |
| --- |

On the dashboard, can you tell me:

1. where you would find the classes and events that are recommended for you
2. where you would find the articles that are recommended for you
3. how you would invite a friend to join PatientCareAnywhere
4. how you would connect with other patients who use PatientCareAnywhere (for patients but also asked of caregivers)
5. where you would find your (or the patient’s) next calendar event
6. where you would find your (or the patient’s) help requests
7. where you would find your messages
8. where you would report symptoms for yourself (or the patient)
9. where you would post updates to wall
10. where you would view wall updates
11. where you would go to view the medical records

On the medical page, can you tell me where you would:

1. find the care team referrals
2. view your (the patient’s) symptom history
3. view your (the patient’s) screening summaries
4. add your (or the patient’s) medical records
5. be able to send a message to the care team member

On the calendar page, can you tell me how you would:

1. add an event to calendar
2. view only the City of Hope’s events
3. find the classes/events/support groups on your calendar

On the Learning Center page, can you tell me where you would:

1. find the articles that are recommended for you
2. find articles about managing pain (navigate the browse function to symptom management)
3. save articles that you like to your favorites section
4. view the articles in your favorites section

On the Support Group page, can you tell me how you would:

1. find support groups about nutrition (browse support groups to nutrition)
2. add the support group to your calendar
3. find more information about the support group

On the Classes and Events page, can you tell me how you would:

1. find events/classes about managing fatigue (browse classes/events to symptom management to fatigue)
2. add the class/event to your calendar
3. find more information about the class/event

On the symptom reporting tool, can you tell me:

1. where you would go if you want to report symptoms (click on the link)
2. what the symptom report instructions are telling you to do
3. how you would report the symptom of pain (or fatigue, depression)
4. what you should click next on the screen (to navigate follow-on questions)
5. what you would click if you wanted to send a note to the care team about this symptom
6. what the color on the symptom you just reported means (symptom report interpret color scores)
7. what you would click if you don’t have any more symptoms to report
8. how would you get to view your symptom summary
9. how you would print your symptom report
10. where you would click if you wanted to return home after viewing this summary
