# Supplementary material for: Usability and Preliminary Efficacy of an Adaptive Supportive Care System for Patients With Cancer: Pilot Randomized Controlled Trial
Source: JMIR Cancer. 2024 Jul 10;10:e49703. doi: 10.2196/49703 (PMC11269963; doi:10.2196/49703)
Supplement: Multimedia Appendix 4 [file cancer_v10i1e49703_app4.docx]

Multimedia Appendix 4. Sample characteristics of included versus excluded pilot randomized controlled trial participants (Phase II)

|  | **Included** (N=59) | **Excluded** (N=13) | **Total** (N=72) |  |
| --- | --- | --- | --- | --- |
|  | N **(%)** | N **(%)** | N **(%)** | ***p*** |
| **Age** |  |  |  | 0.337 |
| Mean (SD) | 53.8 (12.28) | 57.6 (9.76) | 54.4 (11.94) |  |
| Range | (30.0-79.0) | (42.0-68.0) | (30.0-79.0) |  |
| **Gender** |  |  |  | 0.999 |
| Female | 49 (83.1%) | 11 (84.6%) | 60 (83.3%) |  |
| Male | 10 (16.9%) | 2 (15.4%) | 12 (16.7%) |  |
| **Race** |  |  |  | 0.379 |
| Asian | 10 (16.9%) | 0 (0%) | 10 (13.9%) |  |
| Black/AA | 7 (11.9%) | 1 (7.7%) | 8 (11.1%) |  |
| White | 41 (69.4%) | 11 (84.6%) | 52 (72.2%) |  |
| Missing | 1 (1.7.%) | 1 (7.7%) | 2 (2.8%) |  |
| **Ethnicity** |  |  |  | 0.999 |
| Hispanic/Latino | 15 (25.4%) | 3 (23.1%) | 18 (25%) |  |
| Non-Hispanic | 41 (69.5%) | 10 (76.9%) | 51 (70.8%) |  |
| Unknown | 3 (5.1%) | 0 (0%) | 3 (4.2%) |  |
| **Marital Status** |  |  |  | 0.535 |
| Single | 8 (13.6%) | 1 (7.7%) | 9 (12.5%) |  |
| Married | 43 (72.9%) | 11 (84.6%) | 54 (75%) |  |
| Divorced | 6 (10.2%) | 0 (0%) | 6 (8.3%) |  |
| Widowed | 2 (3.4%) | 1 (7.7%) | 3 (4.2%) |  |
| **Education** |  |  |  | 0.999 |
| Less than high school | 3 (5.1%) | 0 (0%) | 3 (4.2%) |  |
| High school | 12 (20.3%) | 2 (15.4%) | 14 (19.4%) |  |
| College | 33 (55.9%) | 8 (61.5%) | 41 (56.9%) |  |
| Graduate school | 11 (18.6%) | 3 (23.1%) | 14 (19.4%) |  |
| **Household Income** |  |  |  | 0.074 |
| Less than $20,000 | 8 (13.6%) | 0 (0%) | 8 (11.1%) |  |
| $20,000-29,999 | 3 (5.1%) | 1 (7.7%) | 4 (5.6%) |  |
| $30,000-49,999 | 6 (10.2%) | 3 (23.1%) | 9 (12.5%) |  |
| $50,000-69,999 | 8 (13.6%) | 0 (0%) | 8 (11.1%) |  |
| $70,000-99,999 | 5 (8.5%) | 4 (30.8%) | 9 (12.5%) |  |
| More than $100,000 | 29 (49.2%) | 5 (38.5%) | 34 (47.2%) |  |
| **Cancer Type** |  |  |  | 0.999 |
| Breast | 43 (72.8%) | 10 (76.9%) | 53 (73.6%) |  |
| Lung | 7 (11.9%) | 1 (7.7%) | 8 (11.1%) |  |
| Prostate | 5 (8.5%) | 1 (7.7%) | 6 (8.3%) |  |
| Missing | 4 (6.8%) | 1 (7.7%) | 5 (6.9%) |  |
| **Stage** |  |  |  | 0.523 |
| 0 | 5 (8.5%) | 2 (15.4%) | 7 (9.7%) |  |
| I | 11 (18.6%) | 2 (15.4%) | 13 (18.1%) |  |
| II | 11 (18.6%) | 4 (30.8%) | 15 (20.8%) |  |
| III | 6 (10.2%) | 1 (7.7%) | 7 (9.7%) |  |
| IV | 15 (25.4%) | 1 (7.7%) | 16 (22.2%) |  |
| Unknown | 11 (18.6%) | 3 (23.1%) | 14 (19.4%) |  |

Abbreviations: M, mean; SD, standard deviation.
